# P-602. Serotype distribution and antimicrobial resistance in pneumococcal disease in Ontario, Canada, before PCV20/21 introduction

**DOI:** 10.1093/ofid/ofaf695.815

**Published:** 2026-01-11

**Authors:** Altynay Shigayeva, Allison McGeer, Huda Almohri, Alyssa Golden, Wayne Gold, Sigmund Krajden, Reena Lovinsky, Irene Martin, Krystyna Ostrowska, Neil Rau, David Richardson, Christie Vermeiren, Zoe Zhong, Christopher Kandel

**Affiliations:** Sinai health, Toronto, Ontario, Canada; Mt. Sinai Hospital, Toronto, Ontario, Canada; LifeLabs, Mississauga, Ontario, Canada; University of Manitoba, Winnipeg, Manitoba, Canada; University of Toronto, Toronto, ON, Canada; Unity Health Toronto, Toronto, Ontario, Canada; Scarborough Health Network, Scarborough, Ontario, Canada; National Microbiology Laboratory (NML), Winnipeg, MB, Canada; Trillium Health Partners, Toronto, Ontario, Canada; Halton Health Care, Toronto, Ontario, Canada; William Osler Health System, Brampton, Ontario, Canada; Shared Hospital Laboratory, Toronto, Ontario, Canada; Sinai Health System, University of Toronto, Toronto, Ontario, Canada; Michael Garron Hospital, Toronto, Ontario, Canada

## Abstract

**Background:**

In Ontario, Canada, the pediatric PCV program changed from PCV13 to PCV15 in 2023, and a publicly-funded PCV20 program for adults started late in 2024. We compare serotype (ST) distribution and antibiotic (AB) resistance in invasive pneumococcal disease (IPD) and respiratory pneumococcal episodes (RPE) prior to the adult program.

**Figure:**
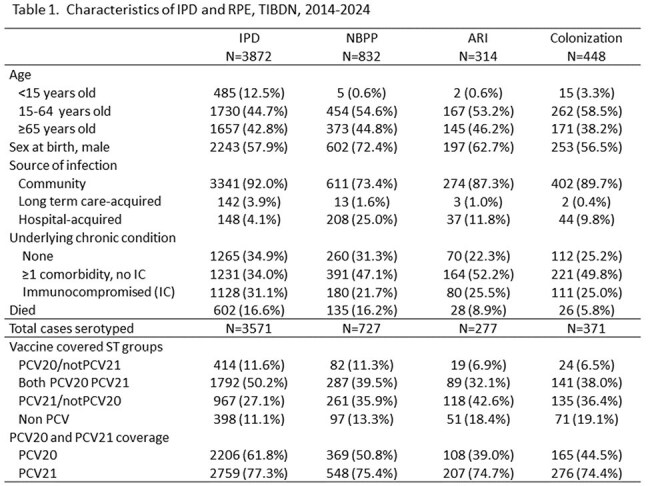

**Figure 1.**
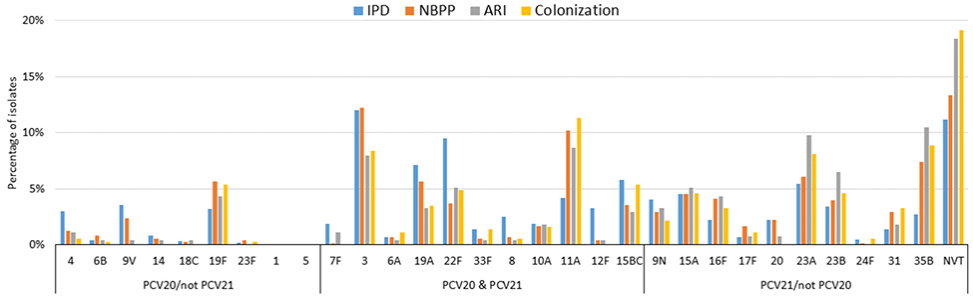
Serotype distribution by diagnostic category, pneumococcal disease in Toronto/Peel hospitals, 2014-2024

**Methods:**

TIBDN performs population-based surveillance for IPD and RPE in Toronto/Peel (pop 4.5M). Microbiology laboratories serving area residents report sterile site (all) and respiratory isolates (hospital labs) of pneumococci; annual audits are performed. Isolates are submitted to a central laboratory for serotyping (ST) and antimicrobial susceptibility testing (AST). RPE is classified as non-bacteremic pneumococcal pneumonia (NBPP), acute respiratory infection (ARI), and colonization (COL).

**Figure 2.**
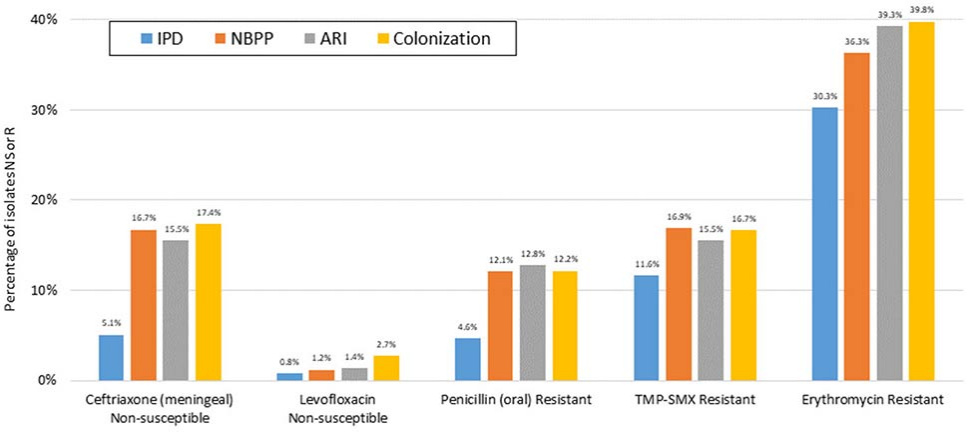
Prevalence of resistant/non-susceptible isolates by diagnostic category, pneumococcal disease, TIBDN, 2014-2024

**Figure 3.**
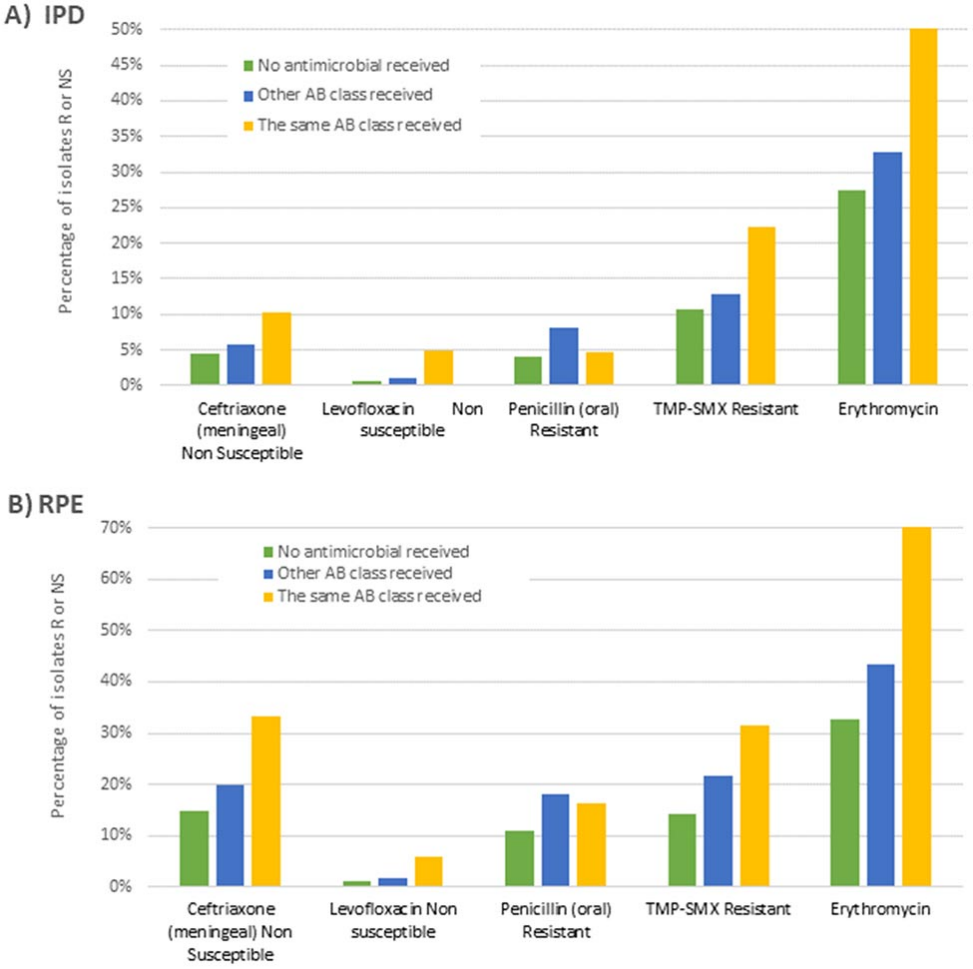
Impact of exposure to antibiotics during the 3 months prior to pneumococcal infection, TIBDN, 2014-2024. Panel A shows results for IPD and Panel B for respiratory pneumococcal episodes

**Results:**

From 2014-2024, 3872 IPD and 2714 RPE cases (832 NBPP, 314 ARI, 448 COL, 1120 unclassified; most due to presence of 2^nd^ pathogen). Patient characteristics are shown in Table 1. Children represented 12.5% of IPD, and < 1% of NBPP and ARI, 3% of COL. Immunocompromise was more common among IPD vs NBPP (31.1% vs 21.7%, *P*< .001). Case fatality was highest among IPD (16.6%) and NBPP (16.2%).

IPD incidence declined during COVID-19 public health interventions, but was similar in 2023/4 and 2015-2019 in all age groups. In 2023/2024, annual incidence was 6.5, 4.7, and 16.6 cases per 100,000 among < 15, 15-64 and ≥65 yr-olds.

ST were available for 90.5% and AST for 91% episodes. ST distribution differed among diagnostic categories (Figure 1). ST 3, 9V, 19A were more common in IPD and NBPP. ST 4, 22F, 8, 12F, 15BC were more common in IPD. ST 11A and 35B were common in RPD vs IPD. PCV21 ST coverage was higher than PCV20 in all diagnostic groups (Table 1).

As compared to IPD, isolates from RPE were more likely to be antibiotic (AB) resistant (Figure 2). Resistance did not differ among RPE categories. In both IPD and RPD, AB resistance was higher among those who had prior exposure to the same class of AB (Figure 3).

**Conclusion:**

IPD incidence has been stable since 2014 (4 years after pediatric PCV13 implementation). NBPP differs from IPD in ST distribution and AB resistance. NBPP diagnosed at or during admission has outcomes similar to IPD. Recent AB use is an important risk factor for AB resistance

**Disclosures:**

Allison McGeer, MD, Merck: Grant/Research Support|Merck: Honoraria|Pfizer: Grant/Research Support|Pfizer: Honoraria|Sanofi: Grant/Research Support|Sanofi: Honoraria

